# **Erratum notice for:** “NOP14 inhibits melanoma proliferation and metastasis by regulating Wnt/β-catenin signaling pathway” [Braz J Med Biol Res 2019;52(1):7952]

**DOI:** 10.1590/1414-431X2021e7952erratum

**Published:** 2022-02-04

Jingrong Li^1,2^, Ruihua Fang^2^, Jianqin Wang^3^, and Liehua Deng^1^

^1^Department of Dermatology, The First Affiliated Hospital of Jinan University, Guangzhou, China

^2^Department of Dermatology, Guangzhou First People’s Hospital, School of Medicine, South China University of Technology, Guangzhou, China

^3^Department of Dermatology, Guangzhou Institute of Dermatology, Guangzhou, China

Correspondence: Liehua Deng: <liehuadeng@126.com>

Erratum for: Braz J Med Biol Res | doi: 10.1590/1414-431X20187952.

The authors notified the Editors of the Brazilian Journal of Medical and Biological Research that there are errors in Table 1 (titles of columns 3 and 4) and that the name of one cell line in the text and in Figures 2, 3, 4, and 5 is incorrect (‘SK-ML110’) in the published article.

The correct cell line in all citations should be ‘SK-MEL-1’ and the correct Table 1 is shown below.

**Table 1:** Correlation between nucleolar protein 14 (NOP14) protein levels and clinicopathological characteristics of patients with melanoma.

Characteristic	n	NOP14 protein levels		P-value
		High expression (++, +++)	Low expression (-, +)	
Age (years)				0.427
<60	21	8	13	
≥60	19	5	14	
Gender				0.919
Male	18	6	12	
Female	22	7	15	
Tumor thickness (mm)				**0.002**
<1	14	9	5	
≥1	26	4	22	
Site				0.427
Sun-exposed	21	8	13	
Sun-protected	19	5	14	
Lymph node metastasis				**0.010**
No	11	7	4	
Yes	29	6	23	

Statistical analyses were carried out with the chi-squared test. Bold type indicates statistical significance (P<0.05).

The correct Figures 2, 3, 4, and 5 are as follows:

**Figure 2:**
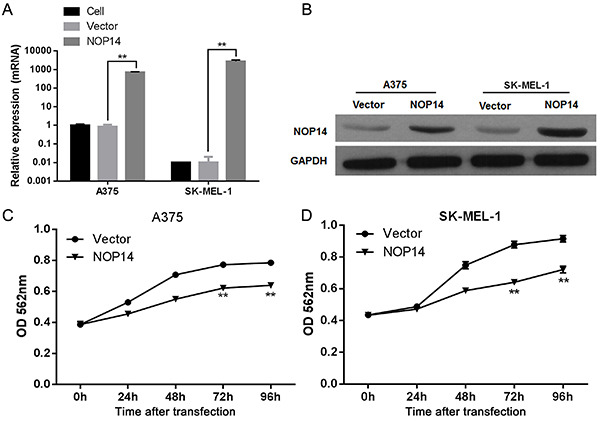
Effect of nucleolar protein 14 (NOP14) overexpression on melanoma cell proliferation. NOP14 mRNA levels (*A*) and protein levels (*B*) in melanoma cell lines transfected with NOP14 overexpression and empty vectors. *C* and *D*, Cell proliferation analysis of melanoma cells after transfection of NOP14 overexpression and empty vectors. Data are reported as means±SD. **P*<*0.01 *vs* empty vector (ANOVA).

**Figure 3:**
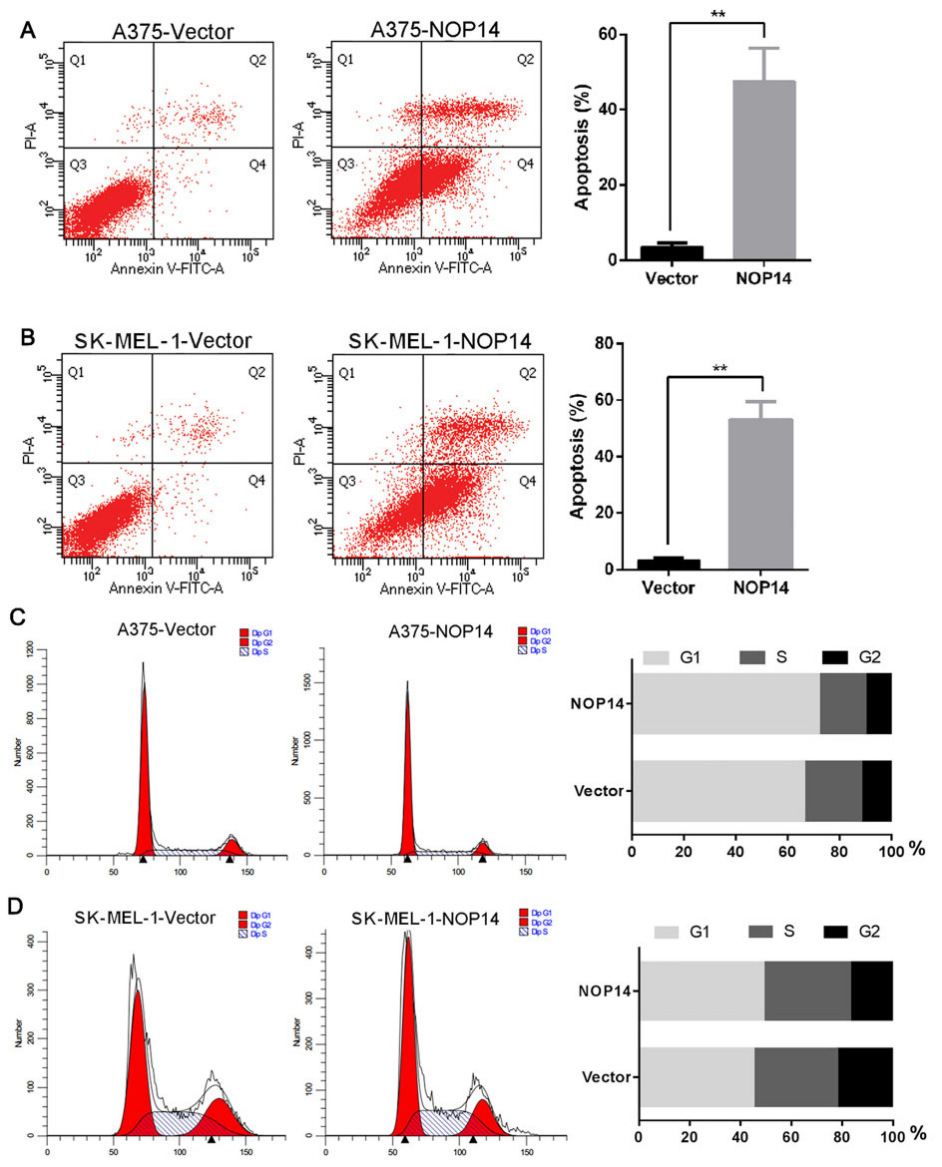
Apoptosis and cell cycle analysis of melanoma cells transfected with nucleolar protein 14 (NOP14) overexpression or empty vector. *A* and *B*, Apoptosis analysis of melanoma cells. *C* and *D*, Cell cycle analysis of melanoma cells. Data are reported as means±SD. **P*<*0.01 *vs* empty vector (*t*-test).

**Figure 4:**
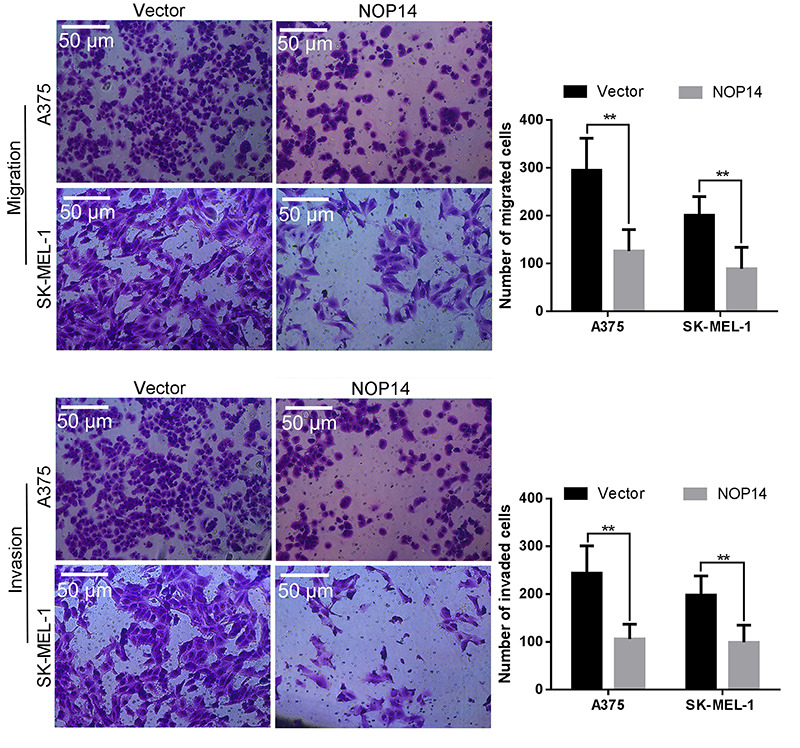
Migratory ability and invasiveness of melanoma cells determined by transwell assay. NOP14: nucleolar protein 14. Scale bar: 50 μm. Data are reported as means±SD. **P*<*0.01 *vs* empty vector (*t*-test).

**Figure 5:**
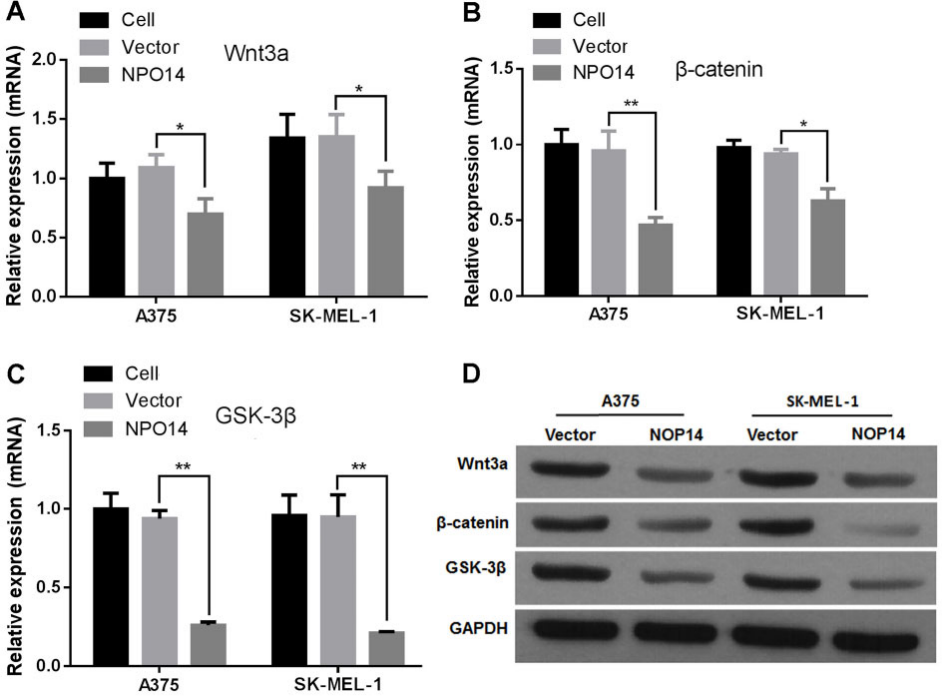
Expression level of Wnt3a, β-catenin, and GSK-3β in melanoma cells. *A* to *C*, Relative expression and *D*, protein levels of Wnt3a, β-catenin, and GSK-3β in melanoma cells transfected with nucleolar protein 14 (NOP14) overexpression and empty vectors. Data are reported as means±SD. *P<0.05, **P<0.01 *vs* empty vector (ANOVA).

